# N1-methyladenosine methylation-related metabolic genes signature and subtypes for predicting prognosis and immune microenvironment in osteosarcoma

**DOI:** 10.3389/fgene.2022.993594

**Published:** 2022-09-06

**Authors:** Guowei Wang, Hongyi Wang, Sha Cheng, Xiaobo Zhang, Wanjiang Feng, Pan Zhang, Jianlong Wang

**Affiliations:** ^1^ Department of Spine Surgery, Third Xiangya Hospital, Central South University, Changsha, Hunan, China; ^2^ Medical College, Hunan Normal University, Changsha, Hunan, China; ^3^ Department of Gastroenterology, Third Xiangya Hospital, Central South University, Changsha, Hunan, China; ^4^ Department of Infectious Disease, Third Xiangya Hospital, Central South University, Changsha, Hunan, China

**Keywords:** N1-methyladenosine methylation, metabolism, osteosarcoma, immune infiltration, immunotherapy

## Abstract

N1-methyladenosine methylation (m^1^A), as an important RNA methylation modification, regulates the development of many tumours. Metabolic reprogramming is one of the important features of tumour cells, and it plays a crucial role in tumour development and metastasis. The role of RNA methylation and metabolic reprogramming in osteosarcoma has been widely reported. However, the potential roles and mechanisms of m^1^A-related metabolic genes (MRmetabolism) in osteosarcoma have not been currently described. All of MRmetabolism were screened, then selected two MRmetabolism by least absolute shrinkage and selection operator and multifactorial regression analysis to construct a prognostic signature. Patients were divided into high-risk and low-risk groups based on the median riskscore of all patients. After randomizing patients into train and test cohorts, the reliability of the prognostic signature was validated in the whole, train and test cohort, respectively. Subsequently, based on the expression profiles of the two MRmetabolism, we performed consensus clustering to classify patients into two clusters. In addition, we explored the immune infiltration status of different risk groups and different clusters by CIBERSORT and single sample gene set enrichment analysis. Also, to better guide individualized treatment, we analyzed the immune checkpoint expression differences and drug sensitivity in the different risk groups and clusters. In conclusion, we constructed a MRmetabolism prognostic signature, which may help to assess patient prognosis, immunotherapy response.

## Introduction

Osteosarcoma (OS) is a primary malignant bone tumour, which derived from mesenchymal cells and occurred mostly in adolescents. Currently, the treatment of osteosarcoma is mainly combined with neoadjuvant chemotherapy before and after surgery. However, over the past three decades, there has been limited improvement in the prognosis of OS (Gianferante et al., 2017). Therefore, the search for biomarkers that allow early diagnosis of OS has become a hot research topic and an imperative in the field of oncology.

Chemical modification of RNA is an important branch of epigenetics, and more than 100 chemical modifications of RNA have been identified (Boccaletto et al., 2018). The common internal modifications in mRNA include N6-adenylation (m^6^A), N1-adenylation (m^1^A), and cytosine hydroxylation (m^5^C) (Han et al., 2021). RNA methylation plays an essential role in almost all steps of mRNA metabolism, and it’s dysregulation is highly correlated with the occurrence and progression of tumours. Aberrant methylation of oncogenes in tumour cells has great potential for early tumour diagnosis.

Metabolic reprogramming, an important feature of tumour cell, is an adaptive change of tumour cells to meet their proliferation and metastasis. Inhibition of tumour cell metabolic processes, including inhibition of glycolysis and amino acid metabolism, is an emerging starvation therapy in recent years (Kerk et al., 2021; Stine et al., 2022). Khodaei et al. (2022) systematically described the generation of effective immunotherapies by regulating the energy metabolism of immune cells. In addition, Lee U. et al. (2022) found that the interaction between metabolic pathways and Hippo signaling pathways could affect the effect of antitumour drugs and drug resistance. A few of studies had reported the potential value of RNA methylation and metabolism-related genes in predicting the prognosis of OS (Liu et al., 2021; Wu Y. et al., 2022; Li et al., 2022). However, it remains to be elucidated whether and how m^1^A regulates metabolism in OS, and the relationship between m^1^A-related metabolic genes (MRmetabolism) and survival in OS has never been explored.

In this study, we analyzed the mRNA expression matrix of OS and normal adipose tissue from the UCSC Xena website to develop a prognosis signature based on two MRmetabolism. We also investigated the correlation of the signature with clinical characteristics, tumour immune microenvironment (TIM) and drug sensitivity.

## Materials and methods

### Data collection

The mRNA expression matrix and clinical data were obtained from the UCSC Xena website (http://xena.ucsc.edu/), including 85 tumour samples and 85 randomized adipose tissue samples. m^1^A methylation genes were obtained from a previous report (Zhang and Jia, 2018). Metabolism-related genes were obtained by c2. cp.kegg.v7.5.1. symbols.gmt, which was downloaded from the GSEA website (http://www.gsea-msigdb.org/gsea/index.jsp).

### Screening m^1^A methylation-related metabolic genes

The “limma” (Wettenhall and Smyth, 2004) and “survival” (van Dijk et al., 2008) packages were used to obtain differentially expressed and prognosis-related metabolic genes and to analyze their correlation with m^1^A methylation genes (|Pearson R| > 0.4 and *p* < 0.05).

### Construction and validation of m^1^A-related metabolic gene signature

Based on the expression profile of MRmetabolism and clinical information, the least absolute shrinkage and selection operator (LASSO) and multivariate Cox (multi-Cox) regression analysis were used to develop a prognostic signature (Bunea et al., 2011). The LASSO regression model was as follows: risk Score = Ʃ [Exp (mRNA) × coef (mRNA)].

Subsequently, we divided all patients into high-risk and low-risk groups with the median value of riskscore in the entire cohort. Next, we randomized all patients into training and test group in a ratio of 3:1. Then, to verify the prognostic ability of the riskscore, Kaplan-Meier (K-M) survival analysis and the time-dependent receiver operating characteristic (ROC) analysis were performed in the whole cohort, training cohort and test cohort, respectively.

### Functional analysis

The curated gene set (kegg.v7.4. symbols.gmt and c5. all.v7.5.1. symbols.gmt) and “clusterProfiler” (Yu et al., 2012) were used to identify significantly enriched pathways between the low-risk and high-risk groups.

### Evaluation of immune cell infiltration and immune checkpoints

We investigated the relationship between riskscore and tumour-infiltrating immune cells (TIIC) by the CIBERSORT algorithm and TIMER2.0 (http://timer.cistrome.org/). The ESTIMATE, immune and stromal scores for the two risk groups were also analyzed. We also investigated the expression levels of immune checkpoints in high-risk and low-risk groups. In addition, the drug sensitivity was calculated in the two risk groups by “pRRophetic” package (Geeleher et al., 2014).

### Consensus clustering based on MRmetabolism

Using the “ConsensusClusterPlus” (Geeleher et al., 2014) package, K-means was applied to cluster patients into two clusters and to further investigate the differences of prognosis, TIIC, immune checkpoint expression and drug sensitivity in the two clusters.

## Results

### Identification m^1^A methylation-related metabolic gene

**FIGURE 1:**
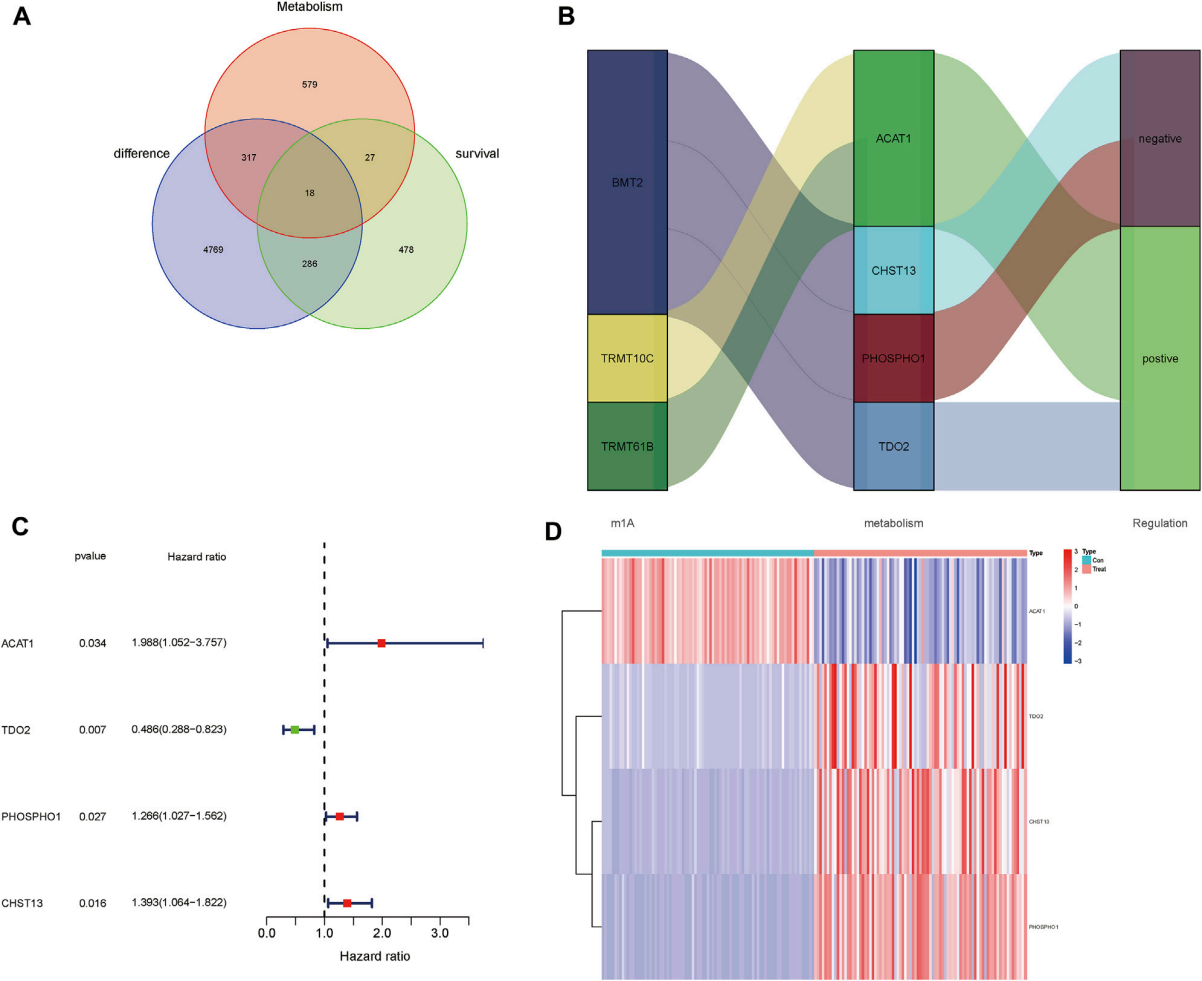
Identification of m^1^A methylation-related metabolic genes. **(A)** The intersection among clusters metabolism-related genes, survival-related genes, and differentially expressed genes. **(B)** The Sankey diagram of m^1^A methylation-related genes and metabolism-related genes. **(C)** The forest plot of four MRmetabolism was plotted by univariate Cox regression analysis. **(D)** The heatmap of differential expressions of four MRmetabolism.

The difference between OS samples and adipose tissue samples was analyzed, we obtained 5,390 differentially expressed mRNAs (|Log ₂ FC| > 1 and *p* < 0.05). Meanwhile, through the survival analysis, we found 809 mRNAs associated with prognosis. Subsequently, through the GSEA website, 941 mRNAs were obtained to be associated with metabolic pathways in OS. By Venn diagram, 18 metabolism-related genes are differentially expressed and correlated with prognosis (Figure 1A). Subsequently, correlation analysis was performed, four MRmetabolism (ACAT1, TDO2, PHOSPHO1, and CHST13) were obtained (|Pearson R| > 0.4 and *p* < 0.05) (Figure 1B). The four MRmetabolism were performed univariate Cox (uni-Cox) regression analysis and the differential expression was visualized as a heatmap (Figures 1C,D).

### Construction and validation of the MRmetabolism signature

**FIGURE 2:**
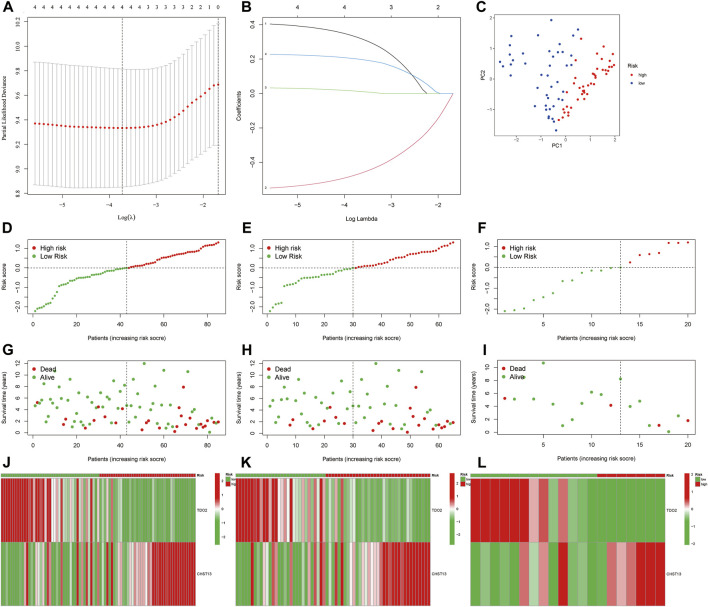
Establishment of prognosis signature. **(A,B)** The LASSO regression model was constructed. **(C)** PCA of OS samples according to the riskscore. **(D–I)** The distribution of the riskscore and survival status of patients in the whole, train and text cohort, respectively. **(J–L)** The heatmap of differential expressions of two MRmetabolism between high-risk and low-risk groups.

Based on the expression of four MRmetabolism in the whole cohort, the following equation was established by LASSO and multi-COX regression analysis (Figures 2A,B): riskscore = (−0.654436269446519* TDO2) + (0.259855036675258* CHST13). We calculated the riskscore for each patient. Then, 85 patients were randomized into the train group (65 samples) and the text group (20 samples) in a ratio of 3:1. Based on the median value of riskscore in the whole cohort, we divided the patients into high-risk and low-risk groups. Principal component analysis (PCA) showed that patients with different riskscore were divided into two parts (Figure 2C). The survival status and riskscore were assessed in the whole, train and text cohort, respectively, (Figures 2D–I). We also analysis the expression of two MRmetabolism in the whole, train and text cohort, respectively, (Figure 2J-L).

**FIGURE 3:**
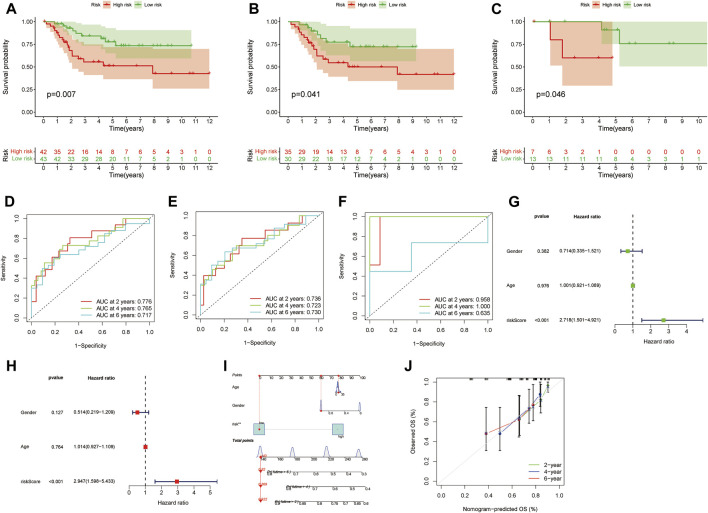
Evaluate the prognostic ability of the signature. **(A–C)** Kaplan–Meier survival estimates of overall survival of patients by the signature in the entire, train and cohorts, respectively. **(D–F)** The entire, train and cohorts of ROC curve analysis, respectively. **(G,H)** Univariate and multivariate analyses the signature. **(I)** A nomogram included clinical features and riskscore for predicting the overall survival of patients with OS at 2-, 4-, and 6-years. **(J)** Calibration curves for 2-, 4-, and 6-years forecasts of nomogram.

Subsequently, we found that the prognosis of low-risk group is better than that of high-risk group by K-M survival in the whole, train and text cohort (Figures 3A–C). The AUCs for 2-, 4-, and 6-year survival were 0.783, 0.766, and 0.712 in the whole cohort, respectively, (Figure 3D). The AUCs for 2-, 4-, and 6-year survival were 0.739, 0.722, and 0.717 in the train cohort, respectively, (Figure 3E). The AUCs for 2-, 4-, and 6-year survival were 0.960, 1.000, and 0.624 in the text cohort, respectively, (Figure 3F). We performed uni-Cox and multi-Cox regression analyses, implying that riskscore, as a high-risk factor, was significantly correlated with overall survival (Figures 3G,H). A nomogram, including clinicopathological variables and riskscore, was also constructed to predict the prognosis of patients at 2, 4, and 6 years (Figure 3I). Calibration curve showed that predicted survival times at 2, 4, and 6 years were consistent (Figure 3J).

### GSEA enrichment analysis

**FIGURE 4:**
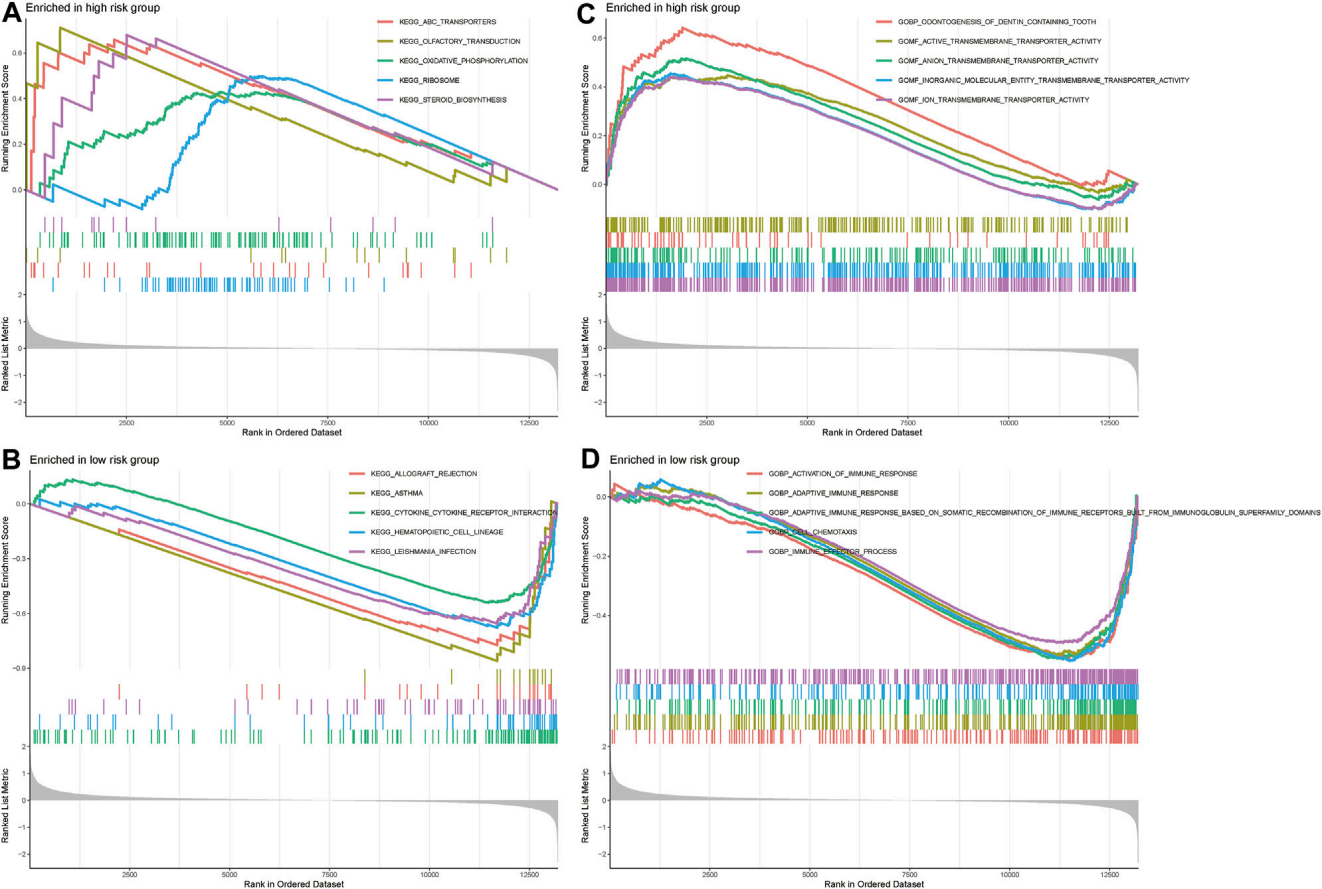
GSEA analysis. **(A,B)** KEGG analysis in the high-risk and low-risk groups. **(C,D)** GO analysis in the high-risk and low-risk groups.

GSEA was used to conduct Kyoto Encyclopedia of Genes and Genomes (KEGG) pathway analysis and Gene Ontology (GO) analysis. ABC transporters signaling pathway, oxidative phosphorylation, ribosome, and steroid biosynthesis were significantly associated with the high-risk group (Figures 4A, B). Cytokine-cytokine receptor interaction, immune effector process, adaptive immune response based on somatic recombination of immune receptors built, adaptive immune response and activation of immune responses were significantly associated with the low-risk group (Figures 4C, D). Thus. we hypothesize that m^1^A may be involved in OS development and progression through immune-related pathways.

### The role of MRmetabolism in tumour immune microenvironment and immunotherapy

**FIGURE 5:**
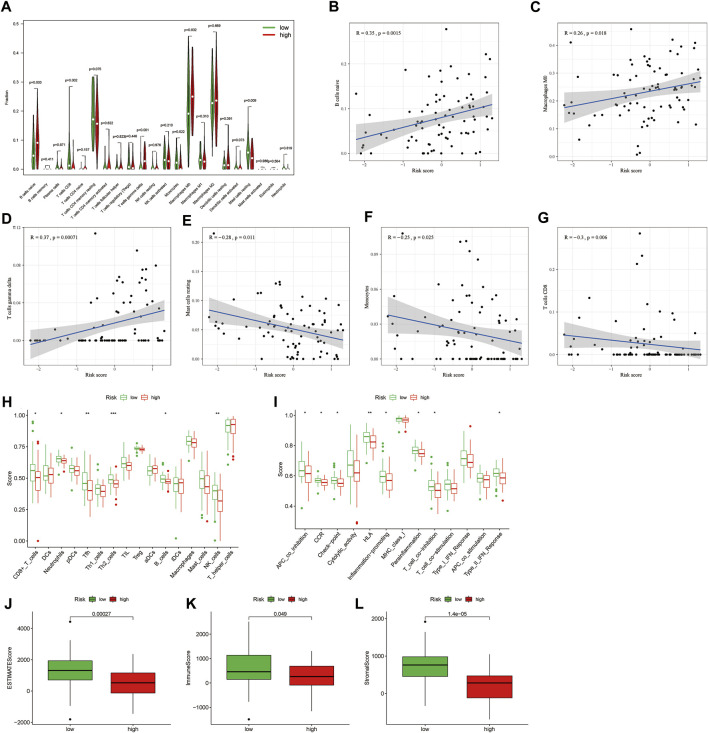
Difference of tumour immune microenvironment between high-risk and low-risk groups. **(A)** The differential infiltration of tumour immune cells between high-risk and low-risk groups. **(B–G)** The correlation between riskscore with immune cell types, including B cells naive, macrophages M0 and T cells gamma delta, mast cell resting, monocyte and CD8 T cell. **(H)** The immune cell bubble of risk groups. **(I,J)** Single-sample gene set enrichment analysis of immune status between low-risk and high-risk groups. **(K–M)** The difference of tumor immune microenvironment (ESTMATE, immune, and stromal score) between high-risk and low-risk groups.

IIC in each sample obtained by “CIBERSORT” algorithm, and then analyze the differences of TIIC in the two risk groups (Figure 5A). We also found that riskscore was positively correlated with B cells naive, macrophages M0 and T cells gamma delta, while was negatively correlated with mast cells resting, monocytes and CD8 T cells (Figures 5B–G). We further analyzed by different immune filtration platforms. Although the algorithms of each platform are different, we can conclude that a large number of immune cells are concentrated in low-risk group (Figure 5H). Then, we explored the relationship between riskscore and immune status by ssGSEA. The results showed that CD8 T cells, neutrophils, Tfh, Th2 cells, B cells, and NK cells were associated with a high degree of infiltration in low-risk group (Figure 5I). APC co inhibition, CCR, check-point, HLA, inflammation-promoting, parainflammation, T cell co inhibition, and Type II IFN reponse were enriched in the low-risk group (Figure 5J). In addition, the low-risk group had higher ESTIMAT, immune and stromal score. All of these indicated that the low-risk group had a higher immune infiltration status (Figure 5K-M). Therefore, we hypothesized that the low-risk group was in an immune activation state relative to the high-risk group.

**FIGURE 6:**
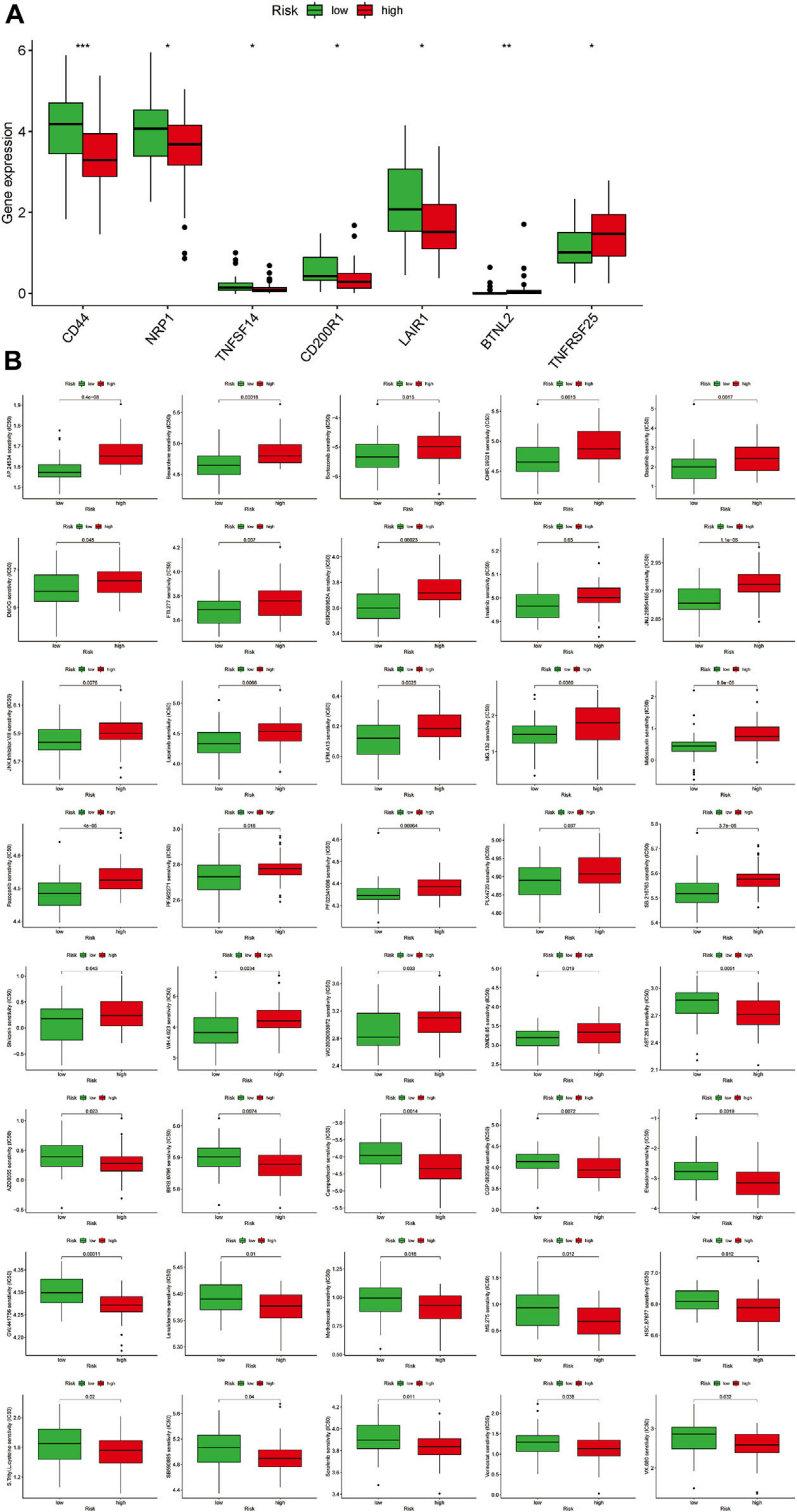
Predict the best immune checkpoint inhibitors and drugs for two risk groups. **(A)** The expression of immune checkpoints in the two risk groups. **(B)** The difference of sensitivity of drugs between high-risk and low-risk groups.

Immune checkpoint inhibitors (ICIs) are an emerging and effective therapeutic strategy for a variety of tumours. While most studies suggest that immune checkpoints are used by tumour cells to evade immune destruction, others suggest that immune checkpoint expression positively correlates with the efficacy of immunotherapy (Marin-Acevedo et al., 2021; Lu et al., 2022). Therefore, we aimed to verify the ability of MRmetabolism in predicting the effective of immunotherapy. The expression of CD44, NRP1, TNFSF14, CD200R1, and LAIR1 was higher in the low-risk group than in the high-risk group (Figure 6A). And BTNL2 and TNFRSF25 were highly expressed in the high-risk group. In addition, we could find that the IC50 of the 40 drugs applied to OS treatment was different between the high and low risk groups (*p* < 0.05) (Figure 6B). This implies that we can select the appropriate immune checkpoint inhibitors and drugs for patients.

### Identification of molecular phenotypes related to MRmetabolism

**FIGURE 7:**
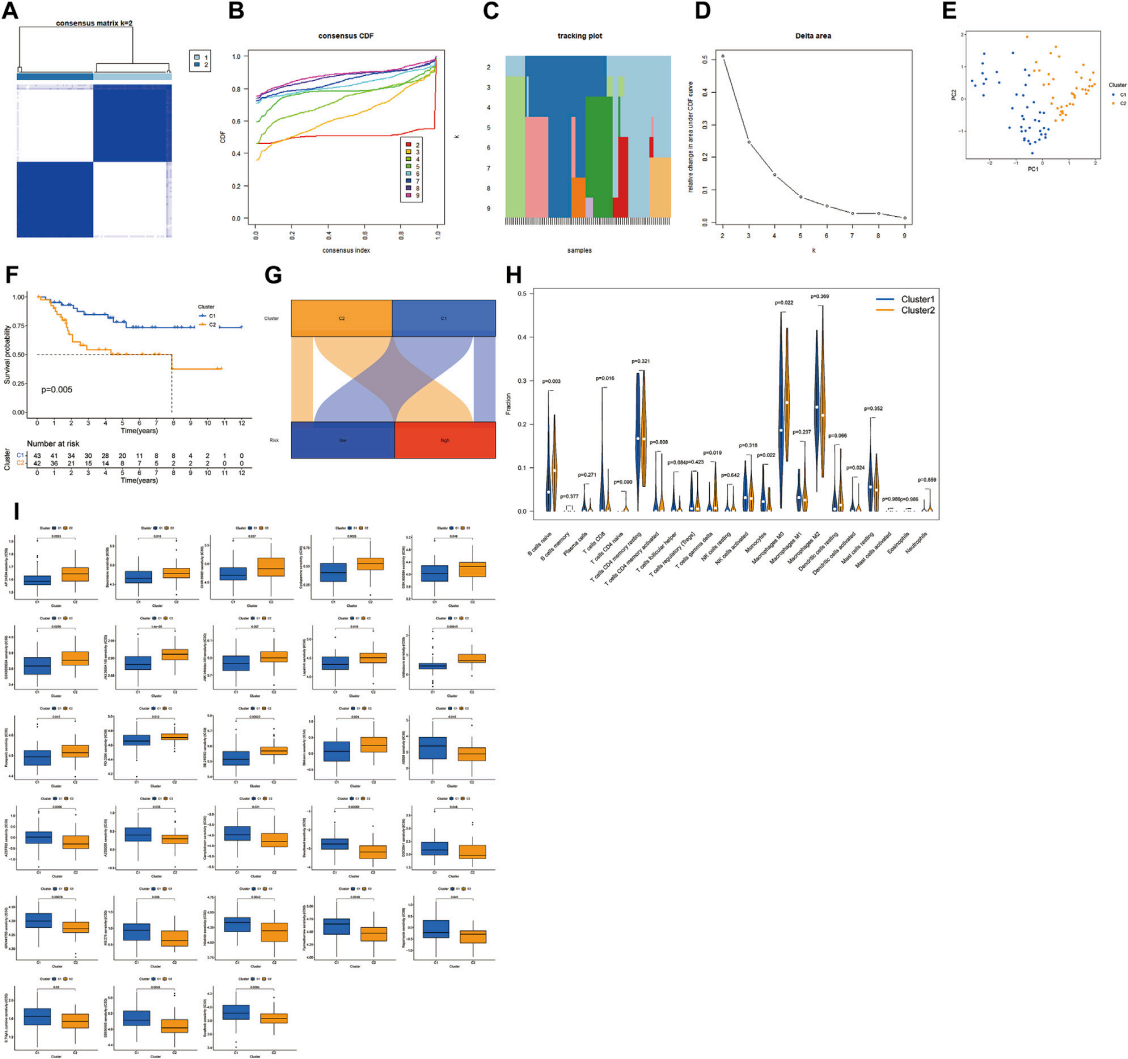
Consensus clustering of two MRmetabolism. **(A)** Consensus clustering matrix for k = 2. **(B)** Consensus clustering CDF with k = 2–9. **(C)** The tracking plot for different k. **(D)** The area under the CDF curve for different k. **(E)** PCA of OS samples according to the clustering. **(F)** The survival estimates of the two clusters. **(G)** The Sankey diagram of the two risk groups and the two clusters. **(H)** The differential infiltration of tumour immune cells in the two clusters. **(I)** The difference of sensitivity of drugs in the two clusters.

Based on the expression profiles of the two MRmetabolisms, we performed consensus clustering. By increasing the clustering variable (k) from 2 to 9, we found that the intra-group correlation is highest and the inter-group correlation is lowest when k = 2 (Figure 7A). Consensus cumulative distribution function (CDF) plots show that the CDF reaches an approximate maximum when k = 2 and the classification is robust (Figures 7B–D). Principal component analysis (PCA) was performed to verify that the two clusters were well differentiated (Figure 7E). K-M survival curve showed that Cluster one patients had a better overall survival than Cluster 2 (Figure 7F). The Sankey diagram showed that most patients with low-risk were Cluster 1, while most patients with high-risk group were Cluster 2 (Figure 7G). The differences of immune cell infiltration in the two clusters showed that B cells naïve, macrophages M0 and T cells gamma delta were highly infiltrated in Cluster 2, while CD8 T cells, monocytes, and dendritic cells activated had a high degree of infiltration in Cluster 1 (Figure 7H). In addition, we found that Cluster 2 had a high ESTIMAT and stromal score. CD44 and VTCN1 were highly expressed in Cluster 1. CD200, CD276, ADORA2A, TNFRSF14, and TNFSF15 were highly expressed in Cluster 2. In addition, we could find that the IC50 of the 28 drugs applied to OS treatment was differential between Cluster one and Cluster 2 (Figure 7I).

## Discussion

Osteosarcoma is the most common primary malignant bone tumour in adolescents. With the rapid changes in science and technology, medical technology is constantly being updated. However, the prognosis of patients with OS has not been greatly improved (Gill and Gorlick, 2021). Untimely early diagnosis and lack of individualized treatment are mainly responsible for the high mortality rate of patients. The identification of reliable biomarkers of sensitivity is essential to improve the prognosis of patients with osteosarcoma.

To maintain the proliferation and metastasis, tumour cells usually undergo metabolic reprogramming (Holbert et al., 2022). In addition, dysregulation between metabolize and immune cells can lead to immune escape of tumour cells (DePeaux and Delgoffe, 2021). Tumour cells preferentially consume glucose and produce lactate through aerobic glycolysis, the latter causing a decrease in the pH of the tumour microenvironment, which in turn hinders cytokine production and T-cell lytic activity (Judge and Dodd, 2020). In addition, lactate can polarize macrophages to a tolerogenic M2-like phenotype (DePeaux and Delgoffe, 2021). Targeting metabolic pathways has been reported to enhance the efficacy of tumour immunotherapy (Wu H. L. et al., 2022b; Khodaei et al., 2022).

m^1^A methylation can affect tumour progression. m^1^A demethylation induced by ALKBH3 can promote protein synthesis in tumour cells (Ueda et al., 2017). The prognosis of breast or ovarian tumour could be affected by the stability of macrophage colony-stimulating factor 1, which was regulated through m^1^A demethylation (Woo and Chambers, 2019). There are few reports about the effects of m^1^A on metabolism-related pathways and tumours. Therefore, we propose to explore the role of metabolism-related genes regulated by m^1^A in OS, which may be a new direction for its treatment.

In our study, we identified the regulatory relationships of three m^1^A genes and four metabolism-related genes in OS. Among them, ACAT1 can promote epithelial mesenchymal transition of tumour cells and sensitivity to chemotherapeutic drugs (Han et al., 2022; Ueno et al., 2022). TDO2 increases glycolysis through activation of the Kyn-AhR pathway to promote tumour cell growth (Lee R. et al., 2022). The migration and invasion of hepatoma cells could be regulated through the Wnt5a pathway (Liu et al., 2022). Subsequently, we screened two RMmetabolism (TDO2 and CHST13) to structure the prognosis signature after LASSO and multi-Cox regression analysis. The results of survival analysis showed that the low-risk group had a better prognosis than the high-risk group, and the riskscore was an independent predictor of OS.

GSEA results showed that the high-risk group was closely associated with ABC transporters, oxidative phosphorylation, ribosome, and steroid biosynthesis. As the upregulation of oxidative metabolism in tumour cells could cause hypoxia and consequently immunosuppression, it has been proposed to improve immune efficacy by inhibiting oxidative phosphorylation (Liu and Curran, 2020; Boreel et al., 2021). Kang et al. (2021) systematically described the mechanism and treatment of ribosomes in tumour and disease. Many malignant and autoimmune diseases can be treated with small molecule inhibitors and monoclonal antibodies by targeting sphingolipid metabolism (Kang et al., 2021). The signaling pathways that inhibit steroid synthesis are potential drug targets for the development of novel tumour immunotherapies (Mahata et al., 2020). What is more, the enrichment function of low-risk group is closely related to immune function.

It has been shown that tumour cells and immune cells have common metabolic requirements and nutritional deficiencies in the tumour microenvironment (Scharping et al., 2016; Renner et al., 2017). Next, we assessed the immune status of the two risk populations. We assessed the immune cell infiltration status of each patient by the CIBERSORT algorithm and ssGSEA. The result showed that the low-risk group could be described as immune activated, while the high-risk group could be described as immunosuppression.

Molecular subtypes have been previously reported to be associated with tumour immunosuppression and microenvironment. Different subtypes have different immune status, resulting in different prognosis and immunotherapeutic response. Therefore, we divided the patients into two groups by the two RMmetabolism. Then, performed K-M survival analysis and immune status assessment, we found that Cluster 1 was in an immune activated state and had a better prognosis compared to Cluster 2.

Finally, we found that the high-risk population was highly sensitive to AZD8055, Camptothecin, Elesclomol, GW.441756, MS.275, S. Trityl.L.cysteine, SB590885, and Sorafenib. Low-risk populations had high sensitivity to AP.24534, Bexarotene, CHIR.99021, GSK269962A, JNJ.26854165, JNK. Inhibitor.VIII, Lapatinib, Midostaurin, Pazopanib, SB.216763, and Shikonin. These findings can be applied in the clinic to improve guidance for individualized treatment.

In summary, we constructed a prognostic model for OS patients based on two RMmetabolism to provide prognostic assessment and immune analysis for OS patients and provide new directions for targeted therapy for OS.

## Data Availability

The datasets presented in this study can be found in online repositories. The names of the repository/repositories and accession number(s) can be found below: https://xenabrowser.net/datapages/, UCSC Xena.
